# Comparative Proteomic Analysis of Roots from a Wild Eggplant Species *Solanum sisymbriifolium* in Defense Response to *Verticillium dahliae* Inoculation

**DOI:** 10.3390/genes14061247

**Published:** 2023-06-10

**Authors:** Liyan Wu, Min Gui, Jiaxun Liu, Jie Cheng, Zhibin Li, Rui Bao, Xia Chen, Yaju Gong, Guanghui Du

**Affiliations:** 1 Horticultural Institute of Yunnan Academy of Agricultural Sciences, Kunming 650205, China; wuliyan_123@sohu.com (L.W.);; 2 School of Agriculture, Yunnan University, Kunming 650500, China

**Keywords:** eggplant, fungus, defense response, proteome, iTRAQ

## Abstract

Eggplant verticillium wilt, caused by *Verticillium* spp., is a severe eggplant vascular disease. *Solanum sisymbriifolium*, a wild species of eggplant that is resistant to verticillium wilt, will be beneficial for genetically modifying eggplants. To better reveal the response of wild eggplant to verticillium wilt, proteomic analysis by iTRAQ technique was performed on roots of *S. sisymbriifolium* after exposure to *Verticillium dahliae*, and some selected proteins were also validated using parallel reaction monitoring (PRM). After inoculation with *V. dahliae*, the phenylalanine ammonia lyase (PAL) and superoxide dismutase (SOD) enzymes and the malondialdehyde (MDA) and soluble protein (SP) of *S. sisymbriifolium* roots all exhibited an increase in activity or content compared with that of the mock-inoculated plants, especially at 12 and 24 h post-inoculation (hpi). A total of 4890 proteins (47.04% of the proteins were from *S. tuberosum* and 25.56% were from *S. lycopersicum* according to the species annotation) were identified through iTRAQ and LC-MS/MS analysis. A total of 369 differentially expressed proteins (DEPs) (195 downregulated and 174 upregulated) were obtained by comparison of the control and treatment groups at 12 hpi, and 550 DEPs (466 downregulated and 84 upregulated) were obtained by comparison of the groups at 24 hpi. The most significant Gene Ontology (GO) enrichment terms at 12 hpi were regulation of translational initiation, oxidation-reduction, and single-organism metabolic process in the biological process group; cytoplasm and eukaryotic preinitiation complex in the cellular component group; and catalytic activity, oxidoreductase activity, and protein binding in the molecular function group. Small molecule metabolic, organophosphate metabolic, and coenzyme metabolic processes in the biological process group; the cytoplasm in the cellular component group; and catalytic activity and GTPase binding in the molecular function group were significant at 24 hpi. Then, KEGG (Kyoto Encyclopedia of Genes and Genomes) analysis was performed, and 82 and 99 pathways (15 and 17, *p*-value < 0.05) were found to be enriched at 12 and 24 hpi, respectively. Selenocompound metabolism, ubiquinone, and other terpenoid-quinone biosyntheses, fatty acid biosynthesis, lysine biosynthesis, and the citrate cycle were the top five significant pathways at 12 hpi. Glycolysis/gluconeogenesis, biosynthesis of secondary metabolites, linoleic acid metabolism, pyruvate metabolism, and cyanoamino acid metabolism were the top five at 24 hpi. Some *V. dahliae*-resistance-related proteins, including phenylpropanoid-pathway-related proteins, stress and defense response proteins, plant–pathogen interaction pathway and pathogenesis-related proteins, cell wall organization and reinforcement-related proteins, phytohormones-signal-pathways-related proteins, and other defense-related proteins were identified. In conclusion, this is the first proteomic analysis of *S. sisymbriifolium* under *V. dahliae* stress.

## 1. Introduction

Eggplant (*Solanum melongena* L.) is a commonly cultivated vegetable worldwide, but it can be severely damaged by infection with *Verticillium* spp. The fungal pathogen insults the plant via its roots (especially its wounded roots), then spreads through the entire plant via the vascular system very quickly. This disease will block the uptake of nutrients and water and finally result in eggplant wilting [1].

Eggplant verticillium wilt results in considerable losses to eggplant production [2], especially in the areas of year-round eggplant production. The spores of pathogens can persist in the soil, even without planting eggplant for many years, and they can continuously create new genetic variations [3]. Some methods for controlling eggplant verticillium wilt have been used, such as chemical and biological control [4], but all these are difficult to restrain and eliminate this disease effectively. Thus, the best method of avoiding pathogen attacks is the utilization of resistant eggplant varieties. 

Many efforts have been made to generate several verticillium-wilt-resistant eggplant materials, but very few cultivars are resistant to verticillium wilt. In previous studies, different conclusions have been made on the inheritance of verticillium wilt resistance in eggplant until now [5,6,7,8,9]. Understanding the molecular mechanisms and molecular components that participated in the eggplant resistance to verticillium wilt will provide vigorous support for preventing this disease.

*S. sisymbriifolium* is a wild eggplant species that has been identified as resistant to verticillium wilt [7,10]. To understand its molecular mechanisms leading to verticillium wilt resistance, RNA Sequencing (RNA-Seq) was once used to analyze and discover the expressed genes of *S. sisymbriifolium* in the defense response to *V. dahliae* inoculation [11]. The previous studies also indicated that several genes involved in the metabolism of lignin, phenylalanine, and phytohormones might play crucial roles during eggplant responses to *V. dahliae* infection [7]. 

As proteins are the core executors of most biological activities, functions, and processes in plants, proteomic analyses for the detection of plant–pathogen interactions can provide new insights into the molecular mechanisms that may be involved in the defense processes [12]. In this study, the iTRAQ (Isobaric Tag for Relative and Absolute Quantitation) technique is used to further identify expressed proteins in *S. sisymbriifolium* roots after *V. dahliae* inoculation. The results will enrich the knowledge of the molecular processes and components that participate in the wild eggplant defense response to *V. dahliae* infection. 

## 2. Materials and Methods

### 2.1. Eggplant V. dahliae

*V. dahliae* strain (QZ-S) with highly toxic pathogenicity was isolated from the roots of diseased eggplants in the Kunming area of China, and it was also identified by the morphological and molecular methods in our previous study [10]. Its culture and preparation methods followed the introduction of our previous report prior to inoculation [10]. 

### 2.2. Plant Materials and Inoculation

After sterilization, the seeds of *S. sisymbriifolium* obtained from the Kunming Institute of Botany, Chinese Academy of Science, were allowed to germinate in a 30 °C growing chamber. Then, the germinated seeds were grown in sterilized soilless growing media and placed in a greenhouse with 25/15 °C (day/night) temperatures. At the stage of two pairs of leaves, the seedlings of *S. sisymbriifolium* were treated by soaking their roots in the spores suspension (1 × 10^7^ spores per mL) for 30 min. Control plants (or mock-inoculated plants) were treated with distilled water in the same way. The roots of the control and treatment plants were all sampled after 0, 6, 12, 24, and 48 h, respectively. For each time point, 15 plants were divided into 3 groups randomly as 3 repeats, and the roots of 5 plants were pooled for each group. Plant samples were frozen in liquid nitrogen immediately after being sub-packaged, then stored at −80 °C for later analysis.

### 2.3. Measurement of Physiological and Biochemical Indexes

The roots (0.5 g) of *S. sisymbriifolium* were ground carefully in liquid nitrogen for enzyme extracts and assays. The enzymes activities of superoxide dismutase (SOD), phenylalanine ammonia-lyase (PAL), the content of malondialdehyde (MDA), and soluble protein (SP) were determined by the method described in a previous report [11]. SOD and PAL enzyme activities were assayed by a Total Superoxide Dismutase Assay Kit (A001-1) and a Phenylalanine Ammonia-lyase Test Kit (A137), respectively. MDA and SP contents were measured using a Malondialdehyde Assay Kit (TBA method) and a Plant Soluble Protein Content Test Kit (A145). All these mentioned assay kits were provided by Nanjing Jiancheng Bioengineering Company. Additionally, all of the indexes were detected using a ultraviolet specrophotometer (DDS-306, Fangzhou Company, Chengdu, China).

### 2.4. Protein Extraction

Proteins were extracted from the control and treatment groups at 12 h and 24 h after *V. dahliae* inoculation (two biological replicates). Approximately 0.5 g of each of roots of the *S. sisymbriifolium* sample were frozen in liquid nitrogen and finely powdered. The powder was dissolved in 1 mL lysis buffer (4% (*w*/*v*) SDS, 0.1 mol·L^−1^ DTT, 0.1 mol·L^−1^ Tris-HCl, and pH 7.6). The mixture was shaken vigorously for 15 min on ice, then centrifuged at 30, 000× *g* for 15 min at the temperature of 4 °C. Five volumes of 10% (*w*/*v*) TCA in acetone were added into the collected supernatant, and the mixture was stored at −20 °C overnight. Then, the supernatant was discarded after centrifugation at 30,000× *g* for 15 min at the temperature of 4 °C. Cold acetone was added to wash the precipitate before the precipitate was placed at −20 °C for more than 30 min. The steps of centrifugation and the precipitate’s washing with cold acetone were repeated three times. After drying, 500 μL lysis buffer was added to the pellet. Then, the mixture was shaken for 15 min on ice. The supernatant was collected by centrifugation at 30,000× *g* for 15 min at the temperature of 4 °C and digested with enzymatic hydrolysis by a filter-aided sample preparation (FASP) method [13]. Then, the enzymatic peptide segments were freeze-dried. 

### 2.5. iTRAQ Labeling

The protein samples were dissolved in 30 μL of TEAB (0.5 mol·L^−1^) and labeled by the iTRAQ^®^ Reagent-8 Plex Multiplex Kit. One unit of iTRAQ reagent was mixed with 70 μL of isopropanol. Two control (mock-inoculated) samples (12 hpi and 24 hpi) were labeled with iTRAQ tags 113 and 114, and two treatment (*V. dahliae*-inoculated) samples (12 hpi and 24 hpi) with tags 117 and 118, respectively. All of the labeled protein samples were incubated at room temperature for 2 h.

### 2.6. LC-MS/MS Analysis

Dry protein samples were resuspended in 100 μL buffer A (25 mmol·L^–1^ NaH_2_PO_4_ at 25% (*v*/*v*) ACN, pH 3.0) and SCX (strong cation exchange), then performed on an HPLC (High-Performance Liquid Chromatography) pump system (Shimadzu LC-20AB). Fractions separation was processed using a nonlinear binary gradient starting with buffer A at a flow rate of 1000 μL·min^–1^ and subsequently transferring to buffer B. All fractions were incubated and dried in a vacuum freeze dryer for LC-MS/MS (Liquid Chromatography—Tandem Mass Spectrometry) analysis. Protein samples were resuspended in 50 μL RPLC (Reversed-Phase Liquid Chromatography) buffer A (0.1% (*v*/*v*) FA, 5% (*v*/*v*) ACN) and were analyzed using an online Eksigent 1D plus combined with a Triple TOF 6600 System (Applied Biosystems, Foster, CA, USA). All data were analyzed with Analyst TF (Applied Biosystems, Foster, CA, USA).

### 2.7. iTRAQ Protein Identification and Quantification

The MS/MS data for protein identification and quantification were performed using ProteinPilot v5.0 (AB Sciex) with the Paragon Algorithm against the plant database from NCBI (National Center for Biotechnology Information). The identified proteins were accepted with a protein confidence index ≥ 95% and the threshold of Unused Prot Score > 1.3. A cutoff value of 1.5-fold was set to identify upregulated and downregulated proteins, and a *p*-value < 0.05 was established for differentially expressed proteins (DEPs).

### 2.8. Bioinformatics Analysis

OmicsBean software (http://www.omicsbean.com:88/, accessed on 25 April 2021) was used to annotate differentially expressed proteins. DEPs were classified and enriched by GO (Gene Ontology) annotations and KEGG (Kyoto Encyclopedia of Genes and Genomes) classifications.

### 2.9. Targeted Protein Verified by Parallel Reaction Monitoring (PRM) Analysis 

To verify the protein expression levels obtained by iTRAQ data, twelve proteins were further selected for validation using the technique of LC-PRM/MS. Protein samples were prepared according to the iTRAQ protocol. The tryptic peptides were separated on an Easy nLC-1200 system (Thermo Scientific, Waltham, MA, USA). PRM analysis was performed on a Q Exactive Plus mass spectrometer (Thermo Scientific, Waltham, MA, USA). The mass spectrometer was operated in the positive ion mode and with the parameters the same as in the experiment of Zhang et al. [14]. The raw data were analyzed to obtain the signal intensities of individual peptide sequences using Skyline 4.1 (MacCoss Lab, University of Washington, Seattle, Washington, USA). For the MS data from PRM analysis, the average base peak intensity of each sample was extracted from the full scan acquisition using RawMeat 2.1 (VAST Scientific). The normalization was also calculated as the methods in the experiment of Zhang et al. [14]. Finally, the relative abundance of protein was defined as the intensity of a certain peptide.

## 3. Results

### 3.1. Analysis of Physiological Indexes in Roots of S. sisymbriifolium

To explore the physiological response of *S. sisymbriifolium* under the stress of *V. dahliae* and determine some key time points for the proteomic analysis, 4 crucial indexes (SOD, PAL, MDA, and SP) were measured at 0, 6, 12, 24, and 48 h post-inoculation (hpi), respectively. 

**Figure 1:**
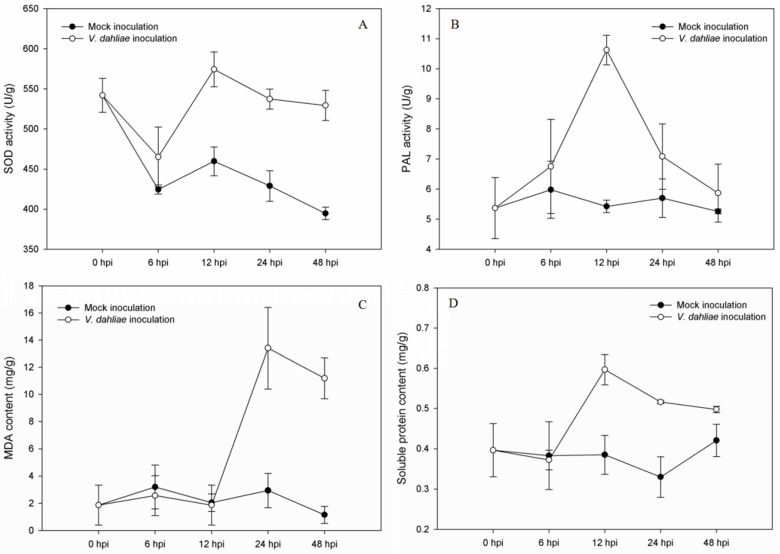
Determination of SOD (**A**), PAL (**B**), MDA (**C**), and soluble protein (**D**) in the roots of *S. sisymbriifolium* measured at 0, 6, 12, 24, and 48 h post-inoculation (hpi) with *V. dahliae* and water (mock inoculation).

After inoculation with *V. dahliae*, the SOD and PAL enzymes activities and the SP content of *S. sisymbriifolium* roots all showed a higher activity or content than the control, especially from 12 to 24 hpi. Additionally, the MDA content appeared to have significant changes at 24 hpi. Thus, all these results indicated that the physiological indexes of *S. sisymbriifolium* changed greatly under the stress of *V. dahliae* exposure, especially at 12 and 24 hpi (Figure 1). 

### 3.2. Proteins Identified by iTRAQ Analysis

**Figure 2:**
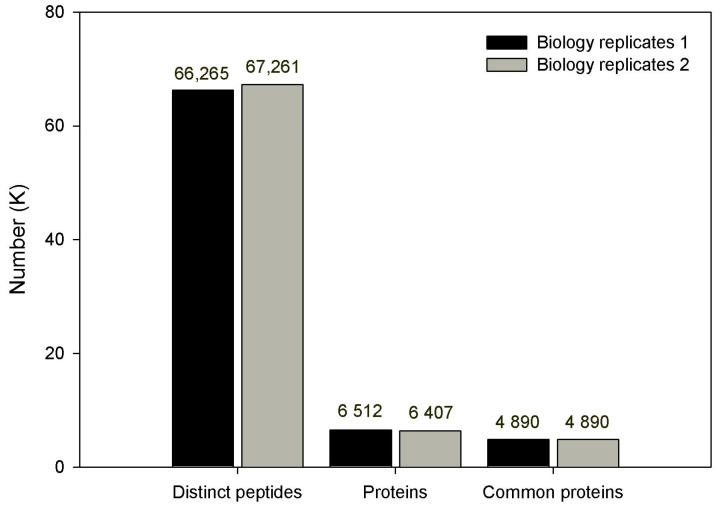
Basic statistics of the identified proteins.

According to the standards of protein identification, a total of 6512 and 6407 proteins were identified from 66,265 and 67,261 peptides (from 2 biological replicates) by searching against a plant database in NCBI. Among them, 4890 proteins were commonly identified in 2 biological replicates (Figure 2 and Table S1).

**Figure 3:**
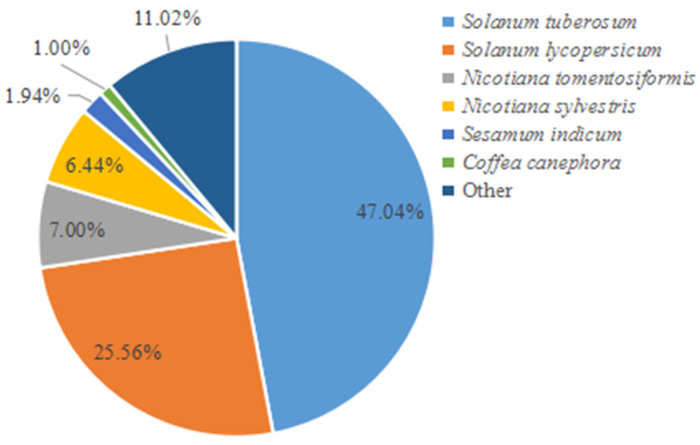
Percentages of the six most abundant annotated species.

Of the 4890 proteins, 4842 proteins (99.02% matching ratio) were successfully annotated by BLAST analysis in NCBI public databases. The species annotation of all matching proteins demonstrated that the first six species annotated accounted for 88.98% of all proteins. Among these annotated species, *S. tuberosum* accounted for 47.04%, and *S. lycopersicum* accounted for 25.56%; they both belong to the *Solanum* spp., and they accounted for 72.60% overall (Figure 3).

### 3.3. Differentially Expressed Proteins (DEPs)

#### 3.3.1. DEPs between Control/Treatment at 12 hpi and 24 hpi

**Figure 4:**
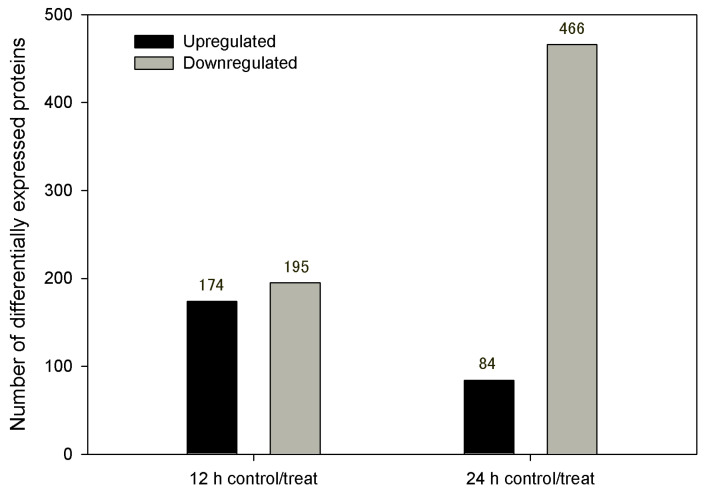
Number of differentially expressed proteins at 12 hpi and 24 hpi.

**Figure 5:**
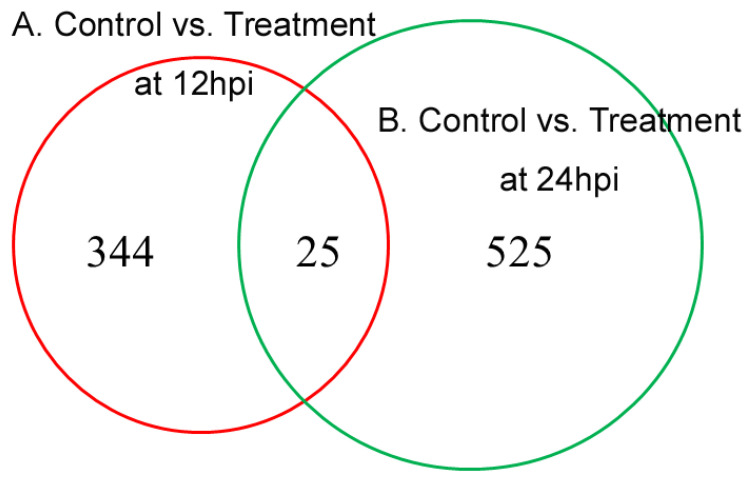
Venn diagram of the differentially expressed proteins between 12 hpi and 24 hpi.

A total of 369 DEPs were identified in the comparison between control and treatment groups at 12 hpi, with 195 being downregulated and 174 upregulated (Figure 4 and Table S2). At 24 hpi, 550 DEPs were identified in the comparison between control and treatment groups, with 466 being downregulated and 84 upregulated (Figure 4 and Table S3). There were 25 overlapping DEPs in the two comparisons by a Venn diagram, with 11 commonly downregulated, 3 commonly upregulated, and 11 first upregulated then downregulated (Figure 5 and Table S4).

#### 3.3.2. Gene Ontology Enrichment Analysis of DEPs

**Figure 6:**
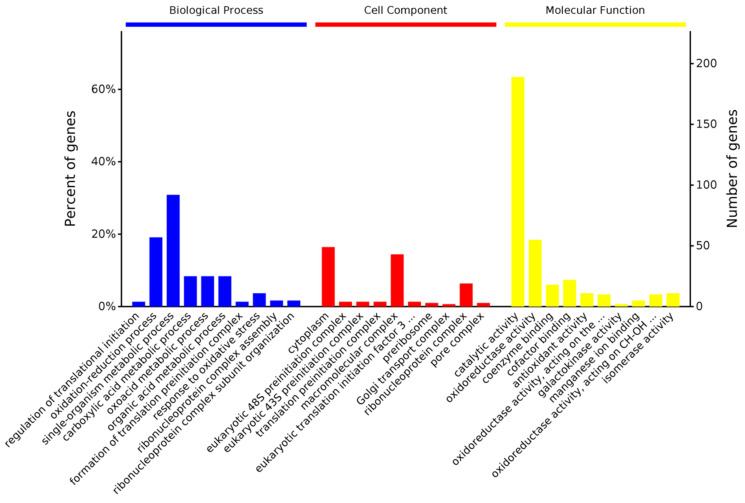
Gene ontology (GO) classifications of the DEPs at 12 hpi.

Gene Ontology (GO) enrichment analysis was performed for 369 and 550 DEPs at 12 and 24 hpi, respectively. In the comparison of the control vs. treatment group at 12 hpi, 369 DEPs were classified into biological processes, cellular components, and molecular function ontology categories, which involved 614, 150, and 376 (86, 26, and 92 (*p*-value < 0.05) terms, respectively (Table S5). We found that the most significant enrichment terms were the regulation of translational initiation (*p* = 1.31 × 10^−4^), oxidation-reduction process (*p* = 1.32 × 10^−4^), and single-organism metabolic process (*p* = 1.39 × 10^−4^), observed with the lowest *p*-value in the biological process group (Figure 6). Correspondingly, most DEPs were enriched in the cytoplasm (*p* = 9.03 × 10^−5^) and eukaryotic preinitiation complex (48S and 43S, *p* = 1.74 × 10^−4^) in the cellular component group (Figure 6). The molecular function group enrichment results indicated that most proteins involved in catalytic activity (*p* = 2.52 × 10^−10^), oxidoreductase activity (*p* = 1.83 × 10^−6^), and protein binding (coenzyme binding (*p* = 3.11 × 10^−5^) and cofactor binding (*p* = 4.50 × 10^−5^)) were DEPs (Figure 6).

**Figure 7:**
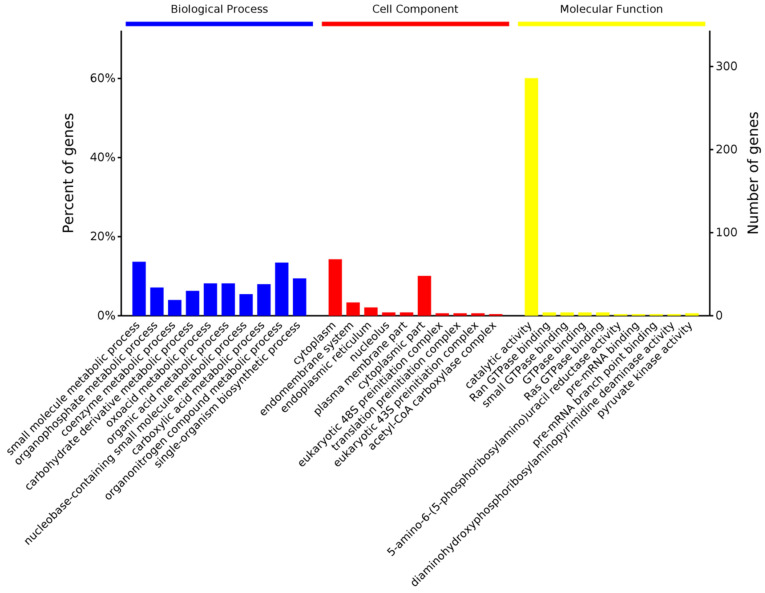
Gene ontology (GO) classifications of the DEPs at 24 hpi.

Among the 550 DEPs in the comparison between the control and treatment group at 24 hpi, 844, 160, and 485 (192, 22, and 87, *p*-value < 0.05) were involved with terms in the biological process, cellular components, and molecular function groups (Table S6), respectively. We found that the most significant enrichment terms were the small molecule metabolic process (*p* = 4.61 × 10^−10^), organophosphate metabolic process (*p* = 2.60 × 10^−7^), and coenzyme metabolic process (*p* = 6.47 × 10^−7^) in the biological process group (Figure 7). Correspondingly, most DEPs were also enriched in the cytoplasm (*p* = 1.18 × 10^−5^), observed with the lowest *p*-value in the cellular component group. The molecular function group enrichment results showed that most proteins involved in catalytic activity (*p* = 1.90 × 10^−7^) and GTPase binding (*p* = 2.08 × 10^−4^) were DEPs (Figure 7).

#### 3.3.3. KEGG Pathway Enrichment Analysis of DEPs

**Figure 8:**
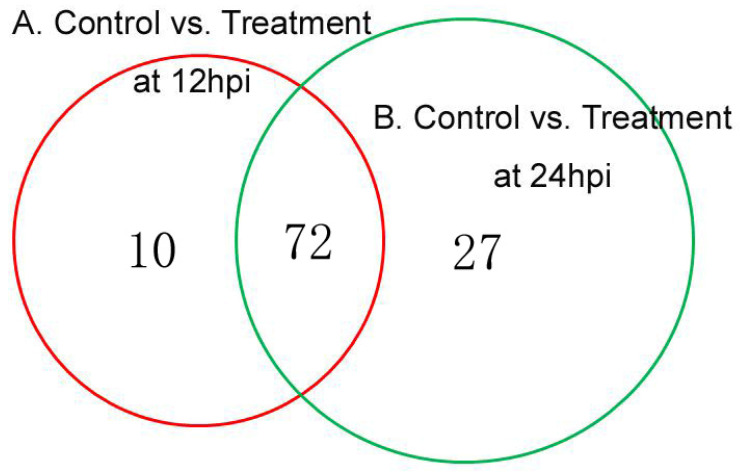
Venn diagram of KEGG pathway enriched between 12 hpi and 24 hpi.

Kyoto Encyclopedia of Genes and Genomes (KEGG) pathway enrichment analysis was performed based on the DEPs identified in the comparison of the control vs. treatment groups. We found 82 and 99 pathways (15 and 17, *p*-value < 0.05) that were enriched at 12 and 24 hpi (Tables S7 and S8), respectively. The top five significant pathways at 12 hpi were selenocompound metabolism (sly00450, *p* = 1.36 × 10^−3^), ubiquinone and other terpenoid-quinone biosynthesis (sly00130, *p* = 2.03 × 10^−3^), fatty acid biosynthesis (sly00061, *p* = 3.16 × 10^−3^), lysine biosynthesis (sly00300, *p* = 4.75 × 10^−3^), and the citrate cycle (TCA cycle) (sly00020, *p* = 6.47 × 10^−3^) (Table 1). At 24 hpi, glycolysis/gluconeogenesis (sly00010, *p* = 8.93 × 10^−5^), biosynthesis of secondary metabolites (sly01110, *p* = 4.46 × 10^−4^), linoleic acid metabolism (sly00591, *p* = 1.40 × 10^−3^), pyruvate metabolism (sly00620, *p* = 4.57 × 10^−3^), and cyanoamino acid metabolism (sly00460, *p* = 4.82 × 10^−3^) were included in the five most significant pathways (Table 2). There were 72 overlapping KEGG pathways in the 2 comparisons by a Venn diagram, but 10 pathways were only present at 12 hpi, and 27 pathways were only present at 24 hpi (Figure 8). In the 72 overlapping KEGG pathways, the DEPs were also differed in their number and regulation mode.

### 3.4. Validation of iTRAQ Data for Selected Proteins by PRM Analysis

**Figure 9:**
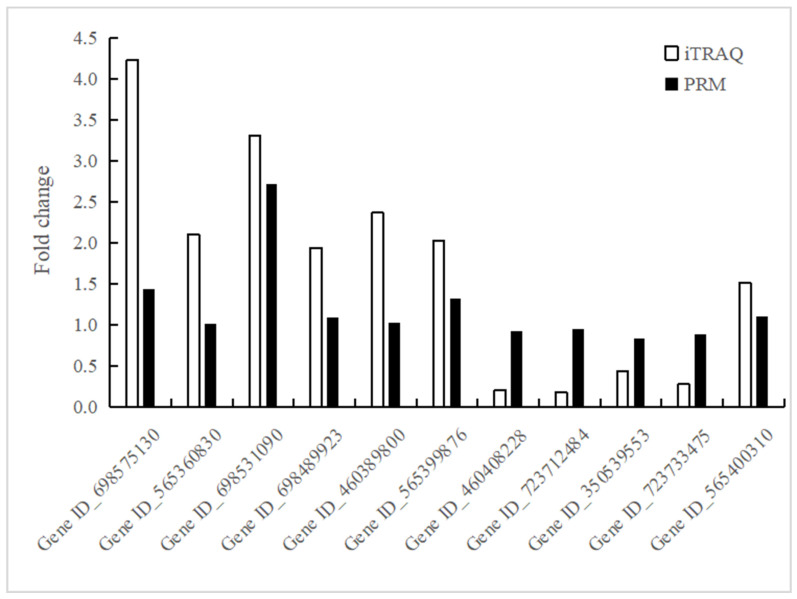
Relative expression levels of selected proteins measured by PRM and iTRAQ.

In this study, the expression levels of some proteins were quantified by LC-PRM/MS analysis to confirm the iTRAQ data. Twelve candidate proteins related to resistance to verticillium wilt were selected for PRM analysis. Among 12 target candidate proteins, 11 proteins were identified as unique peptides with MS/MS spectrum, and they are proteins involved in the defense responses, such as peroxidase, glycine-rich RNA-binding protein, glutathione s-transferase, and auxin-induced protein. In general, the trends in the change in the expression levels of 11 proteins detected by PRM and iTRAQ are basically consistent (Figure 9). However, there are some differences between the values in iTRAQ and PRM data, which may be due to their different detection and analysis methods. 

## 4. Discussion

### 4.1. Wild Eggplant Species S. sisymbriifolium

Breeding verticillium-wilt-resistant eggplant cultivars is the most effective means of controlling verticillium wilt. The fact that the mechanisms of eggplant response to *Verticillium* infection still are unclear and that the existing eggplant cultivars are all not resistant may be why lots of attempts to breed verticillium-wilt-resistant eggplants have not been successful so far. 

Wild eggplant species (or germplasms) have been found to be resistant to *Verticillium*, but most reports have been on *S. torvum*, *S. aculeatissimum*, and *S. linnaeanum* [7,8,15,16]. To better understand the defense response to *V. dahliae*, transcriptome analysis of *S. aculeatissimum* in response to *V. dahliae* infection was performed [7], but only a few *V. dahliae*-resistant-related genes were identified. *S. sisymbriifolium*, the closest wild relative of the domestic eggplant, was found to be highly resistant to *V. dahliae* in our previous study [10]. It could be used as a wild eggplant source of resistance in breeding. However, species hybridization between wild and cultivated eggplants is extremely difficult. Therefore, the molecular mechanisms of resistance in *S. sisymbriifolium* to this eggplant disease (*V. dahliae*) need to be explored. In our previous study, a subset of genes in *S. sisymbriifolium* that may play an important role in activating *V. dahliae* defense responses, including *Ve* and *PR* genes, were screened by RNA-sequencing [11]. However, the function of these genes needs to be further verified. Proteomic identification of differentially expressed proteins could provide new insights into the molecular mechanisms that may be involved in the defense processes of plants to *V. dahliae* infection [17]. Additionally, this is the first study to perform proteomic analysis of *S. sisymbriifolium* roots in defense response to *V. dahliae* inoculation by iTRAQ technology.

### 4.2. Inoculation and Sampling

Studies of DEPs in plants induced by pathogen infection must take internal and external factors into account. The exact material sampled and the time of sample collection are both especially important. As a soil-borne fungus, the spores of *V. dahliae* can enter plant roots and reach the hypocotyl tissues [17]. This process needs some time. Some studies showed that a large number of DEPs had been identified at 24 hpi in cotton [17]. However, there were no previous reports for proteomic analysis on eggplants, so we chose some resistance-related physiological indexes (SOD, PAL, MDA, and SP) to determine the key time points. All indexes changed greatly under the stress of *V. dahliae* exposure, especially at 12 and 24 hpi. 

In terms of sample position selection, there are several sites for sample collection, such as root, hypocotyl, and total seedling [5,6,7,8,9]. Generally, the root becomes an appropriate site for sample collection as it is attached tightly to the pathogen and is the first barrier to infection [18]. In this study, 4890 proteins were successfully identified, and 369 DEPs and 550 DEPs were chosen at 12 and 24 hpi. All these further support the idea that roots are suitable sample materials and 12 and 24 hpi are suitable times for sample collection.

### 4.3. DEPs Analysis at 12 hpi and 24 hpi

In previous reports [5,17], there was only one suitable time for sample collection, which simplified the analysis. However, in our study, we chose two key times for a comparative analysis to not only determine the physiological indexes but also the dynamic analysis of *S. sisymbriifolium* roots in defense response to *V. dahliae* inoculation. In our study, a large number (369 and 550) of DEPs could be identified at the two time points, although there were only a few common DEPs at both times. 

Importantly, from the KEGG analysis, some pathways were sustained processes, such as phenylpropanoid biosynthesis (sly00940), phenylalanine metabolism (sly00360), biosynthesis of secondary metabolites (sly01110), ubiquinone and other terpenoid-quinone biosynthesis (sly00130), and so on. In addition, few stress-resistance-related pathways were only enriched at one time point. For example, phenylalanine, tyrosine, and tryptophan biosynthesis (sly00400); flavonoid biosynthesis (sly00941); and the regulation of autophagy (sly04140) were enriched at only 12 hpi. At the same time, pantothenate and CoA biosynthesis (sly00770), plant–pathogen interactions (sly04626), and plant hormone signal transduction (sly04075) were only enriched at 24 hpi. There were more upregulated proteins at 12 hpi than 24 hpi. 

### 4.4. Proteins Involved in the Defense Responses

#### 4.4.1. Phenylpropanoid-Pathway-Related Proteins 

In plant defense against pathogen infection, the functions of phenylpropanoid compounds range from physical and chemical barriers (preformed or inducible) [19]. Additionally, some phenylpropanoid-pathway-related genes were also identified in the transcriptome analysis of the wild eggplant species *S. aculeatissimum* in response to *V. dahliae* [7]. In this study, a number of proteins were identified that participated in the phenylpropanoid biosynthetic pathway (sly00940). Among these DEPs, there were 12 proteins (6 upregulated and 6 downregulated) at 12 hpi and 17 proteins (3 upregulated and 14 downregulated) at 24 hpi. Among them, suberization-associated anionic peroxidase 1 (TAP1) (Gene ID_565360830) and other peroxidase-related proteins (peroxidase 2, 3, 12, and 27-like) (Gene ID_698575130, 565403661, 697099545, and A0A1U7X9X0, respectively) were significantly upregulated. In previous studies, these proteins had a role in lignin biosynthesis and degradation and the response to pathogen attack [20]. 

#### 4.4.2. Stress and Defense Response Proteins

A variety of stress-response proteins were identified to be upregulated or downregulated after *V. dahliae* inoculation in this study. Regulation of alcohol dehydrogenase (Gene ID_565351204), nucleoredoxin (Gene ID_698531090), and annexin (Gene ID_565362066, 697155779) in *V. dahliae*-inoculated wild eggplant roots might modulate the cellular redox state by maintaining NAD1 homeostasis, thus alleviating oxidative stress [21]. Regulation of some defense response proteins such as superoxide dismutase (Gene ID_698489923), peroxiredoxin (Gene ID_565348344), and glycine-rich RNA-binding protein (Gene ID_698373151, 565399876, 525314229) suggested that components of oxidant protection had an important role in the defense responses of *S. sisymbriifolium* to *V. dahliae* infection. Upregulation of glutathione s-transferase (Gene ID_565343884, 460389800, 723753976, 460405517, 460408228) indicated that GST induction was tightly upregulated in the roots during *V. dahliae* infection. There were also many other stress and defense response proteins (Gene ID_565357907, 460381900, 460401636, 723712484, 565342813, M1CYW5, K4C3G6, and O65740) that are responsive to oxidative stress and defend against *V. dahliae* inoculation. Meanwhile, acetyl-CoA acetyltransferase (Gene ID_M1B976) and replication factor C (Gene ID_697151114), which are negative regulators of the defense response, were downregulated in this study.

#### 4.4.3. Plant–Pathogen Interaction Pathway and Pathogenesis-Related (PR) Proteins

Previous studies showed that genes and proteins in plant–pathogen interaction pathways were very important for plant defense against pathogens [22,23]. In our study, there were some proteins (Gene ID_565390263, H6UM42, 565393831, 350539553, M1A6G8, Q9SIF2, 747067401, 565401383, 565399539, and M1B4B9) enriched in the plant–pathogen interaction pathway at 24 dpi, although no protein was enriched in this pathway at 12 dpi. 

So-called pathogenesis-related (PR) proteins can demonstrate potential antimicrobial activities in vitro, and their accumulation in plants is tightly related to plant defense responses. Many plant PR proteins have been identified during the last decade [24], although a direct functional role in plant resistance responses could not be exhibited for them all. In this study, glucan endo-1, 3-β-glucosidase (PR2), peroxidase (PR9), pathogenesis-related protein 10 (PR10) (Gene ID_51317936), and pathogenesis-related protein STH 2 (Gene ID_698495367) were identified. Importantly, PR10 was also screened by the transcriptome sequencing analysis in our previous results [11].

#### 4.4.4. Proteins Involved in Cell Wall Organization and Reinforcement 

For plants, their cell wall is the first line of defense to protect themselves from invasion by fungal pathogens. During pathogenic insult, cell wall structure and composition are modified to withstand the various physical and/or chemical forces of the pathogens [25]. For example, being inoculated with *Pseudomonas syringae*, the resistant plants showed more callose deposition than the wild-type plants in *Arabidopsis* [26]. In our study, some proteins that participated in callose synthesis (Gene ID_460395564; 565369665) were also identified in *S. sisymbriifolium* inoculation with *V. dahliae*. 

Previous studies have shown that the POD enzyme activity of plants can promote lignin biosynthesis and accumulation and the lignification of infected tissues after infection by pathogens [27]. In this study, many peroxidase-related proteins (Gene ID_698575130; 565403661; 697099545; 460388129; 698481304; 568214345; 460407224; 460388984; 565386497 and 565381207) were also induced after *S. sisymbriifolium* inoculation with *V. dahliae*. Some EXP1 homologous proteins (Gene ID_350539003 and Q9XGI6) were found to be DEPs at 12 hpi. Additionally, EXP1 causes the loosening of plant cell walls, evidently by disrupting noncovalent bonds between cellulose microfibrils and matrix glucans [28]. Some identified DEPs (Gene ID_M1C094, M1CEN9, 565403312, M1CB14, and K4CQM0) have cellulose synthase activity and xyloglucan: xyloglucosyl transferase activity for the organization of the plant cell walls. Pectin acetylesterase (Gene ID_565373459), identified as a DEP in this study, can decrease the degree of acetylation of pectin gels, which alters the physical properties of the primary cell wall in plants [29].

#### 4.4.5. Phytohormones-Pathway-Related Proteins 

Plant hormones play an important regulatory role in plant responses to adverse biotic stresses. Pathogen-induced defense responses of plants depend on the action of phytohormones such as auxin (IAA), jasmonates (JAs), ethylene (ET), and brassinosteroid (BR) [30]. In this study, seven proteins (Gene ID_45738252, 723733475, 698521509, 565353752, 565348592, G8Z292, K4AXQ9, Q6TDU2, K4BPM0, and A0A1U7XEY0) involved in the processes of the signaling pathways of phytohormones were significantly differentially accumulated. In tobacco and *Arabidopsis*, previous reports all revealed the essential importance of phytohormones in the regulation of downstream immune signaling events. After pathogenic insult, plants will synthesize a variety of hormones, which can lead to the activation of distinct sets of defense-related genes [31]. 

In our study, some proteins, such as Gene ID_K4AXQ9, which are involved in the jasmonic acid-mediated signaling pathway and can induce systemic resistance, were upregulated. In addition, some brassinosteroid-mediated signaling pathway proteins (Gene ID_698521509, G8Z292) were also significantly upregulated. Inversely, some proteins (Gene ID_565353752, 565348592) were involved in negative regulation of the gibberellic acid (GA) and abscisic acid (ABA)-mediated signaling pathways. In our study, a key biosynthetic enzyme (1-aminocyclopropane-1-carboxylate oxidase) of ethylene was identified as an upregulated protein (Gene ID_565377174) at 12 hpi and a downregulated protein (Gene ID_565368992) at 24 hpi, suggesting that ethylene was implicated in the signal transduction and the activation of defense responses of *S. sisymbriifolium* against *V. dahliae* infection.

#### 4.4.6. Other Defense-Related Proteins

RNA-binding proteins (RBPs) allow plants to act as post-transcriptional regulators in response to pathogen attacks. They have been implicated in defenses against pathogens [32]. A glycine-rich RNA-binding protein (Gene ID_M1BAC2) was significantly upregulated at 24 hpi. Upregulation of heat shock protein 70 (Gene ID_460401797, 565370175) showed that different protein folding pathways were activated during *V. dahliae’s* invasion into the roots from *S. sisymbriifolium*. In addition, the upregulation of DNAJ protein homolog isoform X2 (Gene ID_565400310) at 12 hpi could have a role in heat shock protein binding in response to stimuli. 

We also found that there were many proteins not classified into defense-related pathways that had a significant level of change after infection. Therefore, these proteins (Gene ID_565370290, 565370290, 697103810, 723737751, 565382409, 565367326, 460389506, 697143251, 565352686, and 697153310) may be new and important proteins that help the wild eggplant *S. sisymbriifolium* to resist *V. dahliae*. Especially, an uncharacterized protein (Gene ID_565367326) predicted as phosphoinositide phosphatase SAC1-like had a high fold change value both at 12 and 24 hpi. It may be a candidate defense-related protein, but we need to verify its function.

## 5. Conclusions

In plant defense against *V. dahliae* infection, a number of defense response proteins in *S. sisymbriifolium* were identified via comparative proteomic analysis. These are phenylpropanoid pathway-related proteins, stress and defense response proteins, plant–pathogen interaction pathway and pathogenesis-related proteins, cell wall organization and reinforcement-related proteins, phytohormone-signal-pathway-related proteins, and other defense-related proteins. In future studies, the function of these candidate proteins will be verified, and the molecular mechanism of the wild eggplant species’ defense response to verticillium wilt will be clarified.

## Figures and Tables

**Table 1:** KEGG pathway enrichment analysis of DEPs at 12 hpi.

No.	Enriched KEGG Pathways (ID)	DEPs		*p*-Value
		Up	Down	
1	Selenocompound metabolism (sly00450)	2	2	1.36 × 10^−3^
2	Ubiquinone and other terpenoid-quinone biosynthesis (sly00130)	3	3	2.03 × 10^−3^
3	Fatty acid biosynthesis (sly00061)	4	2	3.16 × 10^−3^
4	Lysine biosynthesis (sly00300)	1	2	4.75 × 10^−3^
5	Citrate cycle (TCA cycle) (sly00020)	1	5	6.74 × 10^−3^
6	Biosynthesis of secondary metabolites (sly01110)	22	25	1.25 × 10^−2^
7	Fatty acid metabolism (sly01212)	4	3	1.58 × 10^−2^
8	Phenylpropanoid biosynthesis (sly00940)	6	6	1.62 × 10^−2^
9	Ascorbate and aldarate metabolism (sly00053)	1	4	1.89 × 10^−2^
10	Nucleotide excision repair (sly03420)	1	4	2.71 × 10^−2^
11	Phenylalanine metabolism (sly00360)	2	3	2.90 × 10^−2^
12	Monobactam biosynthesis (sly00261)	1	1	3.43 × 10^−2^
13	Glycolysis/Gluconeogenesis (sly00010)	7	1	4.04 × 10^−2^
14	Ribosome biogenesis in eukaryotes (sly03008)	2	4	4.75 × 10^−2^
15	Biosynthesis of amino acids (sly01230)	7	5	4.96 × 10^−2^

**Table 2:** KEGG pathway enrichment analysis of DEPs at 24 hpi.

No.	Enriched KEGG Pathways (ID)	DEPs		*p*-Value
		Up	Down	
1	Glycolysis/Gluconeogenesis (sly00010)	1	16	8.93 × 10^−5^
2	Biosynthesis of secondary metabolites (sly01110)	13	63	4.46 × 10^−4^
3	Linoleic acid metabolism (sly00591)	2	3	1.40 × 10^−3^
4	Pyruvate metabolism (sly00620)	2	9	4.57 × 10^−3^
5	Cyanoamino acid metabolism (sly00460)	2	5	4.82 × 10^−3^
6	Fructose and mannose metabolism (sly00051)	2	7	5.15 × 10^−3^
7	Carbon metabolism (sly01200)	4	18	7.78 × 10^−3^
8	Phenylpropanoid biosynthesis (sly00940)	3	14	1.09 × 10^−2^
9	Amino sugar and nucleotide sugar metabolism (sly00520)	3	10	1.12 × 10^−2^
10	Metabolic pathways (sly01100)	15	97	1.70 × 10^−2^
11	Purine metabolism (sly00230)	1	12	2.12 × 10^−2^
12	Protein processing in endoplasmic reticulum (sly04141)	1	17	2.39 × 10^−2^
13	Alanine, aspartate, and glutamate metabolism (sly00250)	2	4	3.20 × 10^−2^
14	Selenocompound metabolism (sly00450)	0	3	4.14 × 10^−2^
15	Biosynthesis of amino acids (sly01230)	2	15	4.59 × 10^−2^
16	Base excision repair (sly03410)	0	5	4.72 × 10^−2^
17	Nonhomologous end-joining (sly03450)	0	2	4.99 × 10^−2^

## Data Availability

Not applicable.

## Supplementary Materials

The following supporting information can be downloaded at: https://www.mdpi.com/article/10.3390/genes14061247/s1, Table S1: Identified proteins of *S. sisymbriifolium* in response to *V. dahliae* inoculation; Table S2: Differentially expressed proteins identified at 12 hpi; Table S3: Differentially expressed proteins identified at 24 hpi; Table S4: Differentially expressed proteins identified both at 12 hpi and 24 hpi; Table S5: GO annotation of identified proteins at 12 hpi; Table S6: GO annotation of identified proteins at 24 hpi; Table S7: KEGG pathways of identified proteins in the response of *S. sisymbriifolium* to *V. dahliae* at 12 hpi; Table S8: KEGG pathways of identified proteins in the response of *S. sisymbriifolium* to *V. dahliae* at 24 hpi.

## References

[1] Dubery I.A., Meyer R. (1996). Specific binding of a *Verticillium dahliae* phytotoxin to protoplasts of cotton, *Gossypium hirsutum*. Plant Cell Rep..

[2] Daunay M.C., Prohens J., Nuez F. (2008). Eggplant. Vegetables II.

[3] Wang F., Ma Y., Yang C., Zhao P., Yao Y., Jian G., Luo Y., Xia G. (2011). Proteomic analysis of the sea-island cotton roots infected by wilt pathogen *Verticillium dahliae*. Proteomics.

[4] Fradin E.F., Thomma B.P.H.J. (2006). Physiology and molecular aspects of Verticillium wilt diseases caused by *V. dahliae* and *V. albo-atrum*. Mol. Plant Pathol..

[5] Yang L., Jue D., Li W., Zhang R., Chen M., Yang Q. (2013). Identification of MiRNA from eggplant (*Solanum melongena* L.) by small RNA deep sequencing and their response to *Verticillium dahliae* infection. PLoS ONE.

[6] Yang X., Cheng Y.F., Deng C., Ma Y., Wang Z., Chen X., Xue L. (2014). Comparative transcriptome analysis of eggplant (*Solanum melongena* L.) and turkey berry (*Solanum torvum* Sw.): Phylogenomics and disease resistance analysis. BMC Genom..

[7] Zhou X., Bao S., Liu J., Zhuang Y. (2016). *De novo* sequencing and analysis of the transcriptome of the wild eggplant species *Solanum aculeatissimum* in response to *Verticillium dahliae*. Plant Mol. Biol. Rep..

[8] Yang X., Zhang Y., Cheng Y., Chen X. (2019). Transcriptome analysis reveals multiple signal network contributing to the *Verticillium* wilt resistance in eggplant. Sci. Hortic..

[9] Yang X., Zhang Y., Xue J., Liu F., Cheng Y. (2019). Analysis of small RNAs from *Solanum torvum* Swartz by deep sequencing. Trop. Plant Biol..

[10] Wu L.Y., Guo Z.X., Zeng L., Bao R., Li Z.B., Gong Y.J. (2017). Resistance identification of Yunnan wild eggplant resources to Verticillium wilt. J. Plant Genet. Res..

[11] Wu L., Du G., Bao R., Li Z., Liu F., Gong Y. (2019). De novo assembly and discovery of genes involved in the response of *Solanum sisymbriifolium* to *Verticillium dahlia*. Physiol. Mol. Biol. Plant..

[12] Coumans J.V.F., Poljak A., Raftery M.J., Backhouse D., Pereg-Gerk L. (2009). Analysis of cotton (*Gossypium hirsutum*) root proteomes during a compatible interaction with the black root rot fungus *Thielaviopsis basicola*. Proteomics.

[13] Wiśniewski J.R., Zougman A., Nagaraj N., Mann M. (2009). Universal sample preparation method for proteome analysis. Nat. Methods.

[14] Zhang J., Cao J., Geng A., Wang H., Liu H. (2021). Comprehensive proteomic characterization of the pectoralis major at three chronological ages in beijing-you chicken. Front. Physiol..

[15] Fei J., Chai Y., Wang J., Lin J., Sun X., Sun C., Zuo K., Tang K. (2004). cDNA cloning and characterization of the *Ve* homologue gene *StVe* from *Solanum torvum* Swartz. DNA Seq..

[16] Liu J., Zheng Z., Zhou X., Feng C., Zhuang Y. (2015). Improving the resistance of eggplant (*Solanum melongena*) to *Verticillium* wilt using wild species *Solanum linnaeanum*. Euphytica.

[17] Zhao F., Fang W., Xie D., Zhao Y., Tang Z., Li W., Nie L., Lv S. (2012). Proteomic identification of differentially expressed proteins in *Gossypium thurberi* inoculated with cotton *Verticillium dahliae*. Plant Sci..

[18] Mehta A., Magalhães B.S., Souza D.S.L., Vasconcelos E., Silva L., Grossi-de-Sa M., Franco O., da Costa P., Rocha T. (2008). Rooteomics: The challenge of discovering plant defense-related proteins in roots. Curr. Protein Pept. Sci..

[19] Dixon R.A., Achnine L., Kota P., Liu C.J., Reddy M.S.S., Wang L. (2002). The phenylpropanoid pathway and plant defence—A genomics perspective. Mol. Plant Pathol..

[20] Passardi F., Cosio C., Penel C., Dunand C. (2005). Peroxidases have more functions than a Swiss army knife. Plant Cell Rep..

[21] Cheeseman J.M. (2007). Hydrogen peroxide and plant stress: A challenging relationship. Plant Stress.

[22] Dodds P.N., Rathjen J.P. (2010). Plant immunity: Towards an integrated view of plant-pathogen interactions. Nat. Rev. Genet..

[23] Rojas C.M., Senthil-Kumar M., Tzin V., Mysore K.S. (2014). Regulation of primary plant metabolism during plant-pathogen interactions and its contribution to plant defense. Front. Plant Sci..

[24] Ali S., Ganai B.A., Kamili A.N., Bhat A.A., Mir Z.A., Bhat J.A., Tyagi A., Islam S.T., Mushtaq M., Yadav P. (2018). Pathogenesis-related proteins and peptides as promising tools for engineering plants with multiple stress tolerance. Microbiol. Res..

[25] Smit F., Dubery I.A. (1997). Cell wall reinforcement in cotton hypocotyls in response to a *Verticillium dahliae* elicitor. Phytochemistry.

[26] Swain S., Singh N., Nandi A.K. (2015). Identification of plant defence regulators through transcriptional profiling of *Arabidopsis thaliana cdd1* mutant. J. Biosci..

[27] Christensen J.H., Bauw G., Welinder K.G., Montagu M.V., Boerjan W. (1998). Purification and characterization of peroxidases correlated with lignification in poplar xylem. Plant Physiol..

[28] Zenoni S., Reale L., Tornielli G.B., Lanfaloni A.L., Porceddu C.A. (2004). Downregulation of the *Petunia hybrida* a-expansin gene *PhEXP1* reduces the amount of crystalline cellulose in cell walls and leads to phenotypic changes in petal limbs. Plant Cell.

[29] Philippe F., Pelloux J., Rayon C. (2017). Plant pectin acetylesterase structure and function: New insights from bioinformatic analysis. BMC Genom..

[30] Bari R., Jones J.D.G. (2009). Role of plant hormones in plant defence responses. Plant Mol. Biol..

[31] Robert-Seilaniantz A., Grant M., Jones J.D.G. (2011). Hormone crosstalk in plant disease and defense: More than just jasmonate-salicylate antagonism. Annu. Rev. Phytopathol..

[32] Oskar M., Buchwald W., Nawrot R. (2014). Plant defense responses against viral and bacterial pathogen infections. Focus on RNA-binding proteins (RBPs). Herba Polonica.

